# Comparative analysis of apical root resorption induced by different intrusion mechanics: A scanning electron microscopy study

**DOI:** 10.6026/973206300213889

**Published:** 2025-10-31

**Authors:** Siddharth Sonwane, Monica Chaurasia, Purvi Awasthi, Aashu Sharma, Subodh Sunil Chaudhari, Amey Rajesh Bhandurge

**Affiliations:** 1 Department of Orthodontics and Dentofacial Orthopaedics, Mansarovar Dental College, Bhopal, Madhya Pradesh, India; 2 Department of Dentistry, Government Medical College, Satna, Madhya Pradesh, India

**Keywords:** Root resorption, orthodontic intrusion, scanning electron microscopy (SEM), mini-screws, premolars

## Abstract

Root resorption is a frequent iatrogenic consequence of orthodontic tooth movement and this study aims to compare the extent of root resorption induced by different intrusion mechanics, including mini-screws, bite turbos and box loops, using scanning electron microscopy (SEM). The sample included 18 maxillary first premolars extracted from nine orthodontic patients undergoing first premolar extractions. Subjects were randomly assigned to three groups (n = 6 teeth per group). Following extraction, one-third of the root was embedded in acrylic, while epoxy resin replicas of the apical two-thirds were prepared using silicone impressions. The samples were electroplated with silver and examined under SEM (JEOL Ltd., Tokyo, Japan) at 20 kV, with magnifications ranging from x35 to x1000. Within the study limitations, mini-screws and turbos produced more pronounced root resorption than box loop mechanics.

## Background:

Root resorption (RR) is defined as the progressive loss of root structure, which occurs physiologically in primary dentition or pathologically in permanent teeth. In orthodontics, RR is recognized as one of the most frequent and unpredictable adverse effects of treatment. While minor changes may reverse upon withdrawal of orthodontic forces, extensive resorption can compromise long-term tooth survival [1, 2]. The etiology of RR is multifactorial, encompassing genetic, hormonal, nutritional and mechanical factors [3]. Patient-related variables such as age, root maturity and biological response interact with treatment factors including type of tooth movement, magnitude and direction of applied forces and treatment duration [4]. This complex interplay makes clinical prediction of RR challenging. The influence of orthodontic techniques has been widely studied. Mc Nab *et al.* reported no significant differences between the Begg and Tweed techniques [5]. In contrast, other studies identified age and type of movement as significant contributors to resorptive severity [6, 7-8]. Notably, prolonged and force-intensive mechanics, particularly intrusion, are consistently associated with greater resorption risk [1, 2]. Detection of RR has advanced over time. Traditional reliance on casts and two-dimensional radiographs provided limited accuracy [9, 10- 11]. Modern methods such as cone-beam computed tomography (CBCT) and finite element modeling (FEM) offer greater predictive capacity but remain assumption-based [12, 13, 14-15]. Intrusion mechanics play an important role in correcting deep bite, curve of Spee and gummy smile. Loop mechanics distribute forces broadly, bite turbos are employed for anterior bite opening and mini-screws deliver skeletal anchorage for controlled intrusion. Each technique applies forces differently relative to the tooth's center of resistance, potentially influencing both the severity and distribution of RR [16, 17, 18-19]. Scanning electron microscopy (SEM) provides superior resolution, allowing direct visualization of crater-like defects and microstructural changes on root surfaces [20]. Given these considerations, the present study evaluated RR associated with mini-screws, bite turbos and loop mechanics using SEM. The guiding research question was: What is the relationship between the rate and severity of root resorption and the type of orthodontic intrusion technique employed? By comparing three clinically relevant mechanics, this study aimed to clarify the biological cost of intrusion and guide evidence-based selection of orthodontic force systems.

## Methodology:

## Study design and sample:

This *in vitro* study analyzed maxillary first premolars extracted for therapeutic reasons during orthodontic treatment. A total of 51 premolars were collected from 26 patients (20 females, 6 males; mean age: 15.3 years; range: 12.7-20 years). All participants and their guardians provided written informed consent before extraction. Ethical standards were followed as per orthodontic research protocols [1, 2- 3].

## Inclusion criteria:

Patients with bimaxillary protrusion, orthodontic plans requiring first premolar extractions, periodontally healthy teeth with complete root development and voluntary participation.

Exclusion criteria included non-extraction treatment plans and patient refusal.

## Group allocation and intrusion mechanics:

Patients were treated with a pre-adjusted Edgewise appliance system [4]. One month after bonding, intrusive forces were applied. Using random allocation, the premolars were divided into three groups (n=17 each):

## Group I (Box loops):

Intrusion with 0.017 x 0.025" TMA box loops, activated monthly for six months.

## Group II (Bite turbos):

Intrusion with bonded turbos, widely used for anterior bite opening in deep bite correction [5, 6].

## Group III (Mini-screws):

Intrusion with stainless steel mini-screws typically employed in gummy smile correction and combined intrusion-retraction mechanics [7, 8]. At the end of six months, the premolars were extracted for microscopic analysis.

## Preparation for SEM analysis:

Extracted teeth were cleaned in 5% sodium hypochlorite for four hours to remove organic debris and rinsed with phosphate-buffered saline. Each crown and coronal one-third of the root was embedded in acrylic blocks. Silicone impressions of the apical two-thirds were prepared, followed by epoxy resin replicas. The samples were silver-coated and examined under a scanning electron microscope (SEM) (JEOL JSM 63100, Japan) at 20 kV. Images were obtained at x35, x100, x500 and x1000 magnifications [9, 10-11].

## Measurement of root resorption:

Resorption sites were identified on mesial and distal root surfaces. All images were calibrated against the SEM scale bar (0.5 mm = 25 mm). Measurements were repeated three times and averaged.

## Length of resorption:

Distance between the most coronal and apical extent of resorption craters.

## Depth of resorption:

Distance from crater base to cementum surface, measured with calibration [12, 13].

## Qualitative grading:

Severity of resorption was assessed using the modified Malmgren scoring system (0-4), adapted for SEM images [14].

## Statistical analysis:

All data were analyzed with ANALYSTAT Version 17.0 (StataCorp, USA). Root resorption variables across the three groups were compared using one-way ANOVA. Post hoc comparisons were performed following normality testing with the Shapiro-Wilk test. Statistical significance was set at p < 0.0001 [15, 16- 17].

## Results:

The mean age of participants was 15.5 years. All patients completed the treatment phase without complications. Intergroup comparisons of mean resorption values demonstrated statistically significant differences across the three mechanics (p < 0.0001). Root resorption severity was assessed using the modified Malmgren scoring system [14]. Of the 51 examined premolars, 97% exhibited resorption scores between 0 and 3. In Group I (box loops), 42.3% of teeth scored between 0 and 1. In Group II (bite turbos), 47.3% scored between >1 and <2, while in Group III (mini-screws), 7.4% of teeth scored between >2 and 3. The correlation between overall scores and group-specific values was strong and statistically significant (r = 0.71, p = 0.0001). The apical third was the most frequently affected site, whereas the gingival third was the least involved, consistent with previous reports [1, 2 and 6]. Group-specific distributions were Group I (box loops): Apical third 2.5%, middle third 22.7%, gingival third 0%; Group II (turbos): Apical third 32.2%, middle third 18.9%, gingival third 18.9%; and Group III (mini-screws): Apical third 43.3%, middle third 32.2%, gingival third 21.1%. These differences were statistically significant (p = 0.0001). Marked variation in the length and depth of resorption was observed among groups. The severity of defects increased progressively from Group I → Group II → Group III and from the gingival third → apical third of the root surface.

Group I: At x100 magnification, irregular concavities extended from the apical to the middle third; no changes were observed in the gingival third. At x500, multiple shallow lacunae were visible at the apex. At x1000, resorption appeared as discrete scooped-out craters. Group II: At x100, resorption extended from the apex to the gingival third, with blunt apices and crater-like irregularities in the middle third. The gingival third demonstrated granular, eggshell-like roughness. At x500, alternating intact cementum and resorption craters were noted near the apical foramen. At x1000, funnel-shaped defects extended toward the apex. Group III: At x100, widening of the apical foramen and radiating resorptive activity from the apex were evident. At x500, surface topography was ragged and irregular. At x1000, sharp, cut-like apical borders were observed with clear demarcation between affected and unaffected cementum. These findings confirmed the more destructive nature of intrusive forces delivered close to the center of resistance, particularly with mini-screw mechanics [7, 16]. Resorption extending into dentin was observed in 46.7% of the total sample. Group distribution was Group I: 11.4% (6 teeth), Group II: 18.4% (8 teeth), Group III: 21.7% (10 teeth). Involvement was most frequent in the disto-apical and mesio-apical regions, especially in Groups II and III. These findings align with prior studies indicating that force magnitude and duration directly influence the likelihood of dentinal invasion [6, 8 and 17].

## Discussion:

Orthodontically induced root resorption (OIRR) is a complex biologic process influenced by mechanical, physiologic and genetic factors. Although minor resorption is a common finding during orthodontic therapy, extensive apical loss remains a significant concern as it compromises tooth prognosis [3]. Intrusion mechanics, in particular, have been consistently associated with higher resorption risk due to the concentration of forces near the root apex [5]. In the present study, root resorption was detected in all groups, with severity increasing from box loops → bite turbos → mini-screws. These findings corroborate earlier reports showing that force magnitude, direction and proximity to the center of resistance are decisive in the extent of resorption [7, 8- 9]. Box loops (Group I) demonstrated the least resorption, likely due to broader force distribution and dissipation away from the center of resistance [10]. In contrast, bite turbos (Group II) and mini-screws (Group III) produced more localized, severe defects. SEM findings included blunt apices and funnel-shaped defects with turbos and widened foramina with sharp apical cuts in mini-screw cases. Similar patterns have been described by Kvam [20] and Pandis *et al.* [16], who observed that intrusive mechanics delivering concentrated forces near the apex produce deeper and more extensive craters. The present findings highlight that skeletal anchorage, while biomechanically efficient, may increase biological cost if not carefully calibrated. Mini-screw-related resorption has been linked to high stress concentrations transmitted directly to the apical third [6, 7]. Traditional two-dimensional radiography underestimates early resorption, as lesions <1-2 mm is often undetectable [11]. In contrast, SEM provides high-resolution surface detail and reveals crater-like defects as small as 0.003 mm in the current study. This sensitivity aligns with findings by Durack *et al.* [17] and Ndemuweda *et al.* [18], who emphasized that SEM and CBCT outperform radiographs in identifying early apical resorption. Thus, SEM allowed precise characterization of resorption morphology and depth across mechanics. Although the majority of samples demonstrated scores between 0 and 2 on the modified Malmgren scale, dentinal invasion was observed in nearly half of the specimens, particularly in the mini-screw and turbo groups. This suggests that, beyond cemental changes, deeper structural compromise may occur under high-intensity intrusive forces. Hendrix *et al.* [9] similarly reported increased risk of severe apical shortening in posterior teeth subjected to prolonged intrusion. From a clinical standpoint, these findings underscore the need to balance the efficiency of intrusion with the risk of root compromise. Shorter activation periods, lighter forces and regular monitoring may mitigate biologic damage [2, 12 and 13]. When skeletal anchorage is used, forces should be carefully adjusted and CBCT or digital monitoring may aid in early detection of progressive resorption. The results align with earlier studies reporting that resorption severity is directly related to the magnitude and duration of intrusive forces [8, 14 and 15]. Burstone [10] and Ramirez-Echave *et al.* [11] also noted that apical third regions are most vulnerable; consistent with the current observation that resorption was most frequent in the apical and middle thirds. Moreover, FEM studies by Tanne *et al.* [12] and Barros *et al.* [15] support the hypothesis that stress concentration in the apical region during intrusion correlates with observed resorptive patterns. This study was limited by its *in vitro* design, which may not fully replicate the dynamic reparative processes occurring *in vivo*. Furthermore, the sample size per group was modest and findings may not be generalizable across different age groups or tooth types. Nonetheless, SEM provided detailed insights into microstructural changes, offering a valuable foundation for future longitudinal and clinical investigations.

## Conclusion:

Apical root resorption associated with three commonly used intrusion mechanics-box loops, bites turbos and mini-screws-using high-resolution SEM analysis is shown. Root resorption was observed in all groups, with severity greatest in mini-screws, followed by bite turbos and least with box loops.
